# Transparency and precision in the age of AI: evaluation of explainability-enhanced recommendation systems

**DOI:** 10.3389/frai.2024.1410790

**Published:** 2024-09-05

**Authors:** Jaime Govea, Rommel Gutierrez, William Villegas-Ch

**Affiliations:** Escuela de Ingeniería en Ciberseguridad, FICA, Universidad de Las Américas, Quito, Ecuador

**Keywords:** recommendation systems, explainability in AI, transparency and trust in AI, machine learning, artificial intelligence

## Abstract

In today’s information age, recommender systems have become an essential tool to filter and personalize the massive data flow to users. However, these systems’ increasing complexity and opaque nature have raised concerns about transparency and user trust. Lack of explainability in recommendations can lead to ill-informed decisions and decreased confidence in these advanced systems. Our study addresses this problem by integrating explainability techniques into recommendation systems to improve both the precision of the recommendations and their transparency. We implemented and evaluated recommendation models on the MovieLens and Amazon datasets, applying explainability methods like LIME and SHAP to disentangle the model decisions. The results indicated significant improvements in the precision of the recommendations, with a notable increase in the user’s ability to understand and trust the suggestions provided by the system. For example, we saw a 3% increase in recommendation precision when incorporating these explainability techniques, demonstrating their added value in performance and improving the user experience.

## 1 Introduction

Today, recommender systems have become indispensable tools that mediate our daily interactions with various platforms, from entertainment and social media to e-commerce. Its ability to filter and personalize information has transformed how users discover content and products, adjusting to their preferences and behaviors. However, as these systems advance in complexity, significant challenges related to transparency and trust arise, marking a critical need to address explainability within artificial intelligence (AI).

This research is relevant because it can improve the interface between advanced technology and end users. By exploring how explainability in recommender systems can strengthen transparency and foster trust, the study addresses a central concern for both the scientific community and the public (Amann et al., 2020). The research questions focus on determining the impact of integrating explainability techniques on recommender systems’ precision and user perception.

The literature review reveals a growing interest in AI explainability, with previous research highlighting the benefits and challenges inherent in implementing transparent and understandable systems (Boruah et al., 2023). Although significant progress has been made, gaps remain in how explainability can be optimized to improve system effectiveness and user experience simultaneously. This study aims to fill these gaps, contributing to the existing body of knowledge by empirically evaluating the effects of different explainability methods on recommender systems (Ehsan et al., 2021; Reddy and Kumar, 2023).

In terms of methodology, this study employs a quantitative approach to evaluate the precision and user perception of recommender systems enhanced with explainability techniques. We use MovieLens and Amazon datasets to develop and test recommendation models that integrate methods such as Local Interpretable Model-agnostic Explanations (LIME) and Shapley additive Explanations (SHAP) (Kaneko, 2023; Swathi and Challa, 2023). The choice of this methodology is justified by its ability to provide objective and replicable evaluations of the impact of explainability on system performance and acceptance (Fares et al., 2023).

The results indicate that the integration of explainability improves the precision of the recommendations and significantly increases user satisfaction and trust in the system. These findings underscore the importance of explainability in building recommender systems that are not only technically competent but also transparent and accessible to users. By providing empirical evidence of its benefits in system performance and user experience, this study makes valuable contributions to the existing literature. It lays the foundation for future research in transparent and user-centered AI.

## 2 Literature review

In recommender systems, the literature spans widely on various methodologies, applications, and challenges, ranging from classical approaches to recent advances in machine learning and explainability. Recommender systems have evolved significantly since their inception, as seen in early research by Vesin et al. (2013), who introduced the concept of collaborative filtering. This approach, which exploits user rating patterns to predict preferences, has been instrumental in developing personalized recommendation platforms. However, as data sets have grown in complexity, the literature has highlighted limitations regarding scalability and precision, as Chen et al. (2023) discussed in their studies on matrix decomposition for recommendation improvement.

The incorporation of deep learning techniques in recommendation systems has achieved significant advancements, as shown by works such as Kaddoura et al. (2022) on YouTube have shown how deep neural networks can capture complex non-linear relationships and significantly improve the relevance of recommendations. However, as performance improves, the challenge of explainability arises, as noted in the critical review by Wilming et al. (2022), who emphasizes the need for accurate but interpretable and reliable systems.

Explainability in recommender systems has become an active research frontier, highlighting the importance of offering users a clear understanding of how recommendations are generated. Kartik et al. (2023) introduced the LIME model, a methodology that allows complex models to be interpreted by perturbing the input and observing prediction changes. Similarly, Pai et al. (2022) proposed SHAP, which assigns each feature an importance value based on game theory, allowing for a fairer and more consistent interpretation of feature contributions.

Beyond explainability, the importance of visualization in interpreting large data sets has been recognized. Techniques such as Principal Component Analysis (PCA) and t-distributed Stochastic Neighbor Embedding (t-SNE), discussed in works by Ali et al. (2021) and Lee et al. (2023) respectively, have been highlighted as powerful tools for dimensionality reduction and visualization of high-dimensional data, allowing researchers and users to discern underlying structures and patterns in data interactions.

## 3 Materials and methods

The central purpose of this research is to apply and evaluate explainability techniques in recommender systems to identify and understand how these advanced technologies generate their specific suggestions for users. This study is part of the growing interest in transparency and accountability in AI systems, where the ability to Peng et al. (2022) recommendations become crucial in fostering user trust and adoption of technology. By implementing explainability methods, we seek not only to improve the interpretation of AI systems’ recommendations but also to increase the accessibility and acceptance of these technologies by end users.

For the analysis, two prominent datasets in the field of recommender systems were selected: MovieLens and the Amazon product review dataset. Containing millions of user-provided movie ratings, MovieLens is used to explore recommendations in the context of entertainment and film. On the other hand, the Amazon product review dataset offers a comprehensive view of consumer purchasing behavior and preferences in a diversified e-commerce environment. These data sets are ideal for investigating how explainability techniques can be applied and improve the understanding of recommendations in different domains.

**Figure 1:**
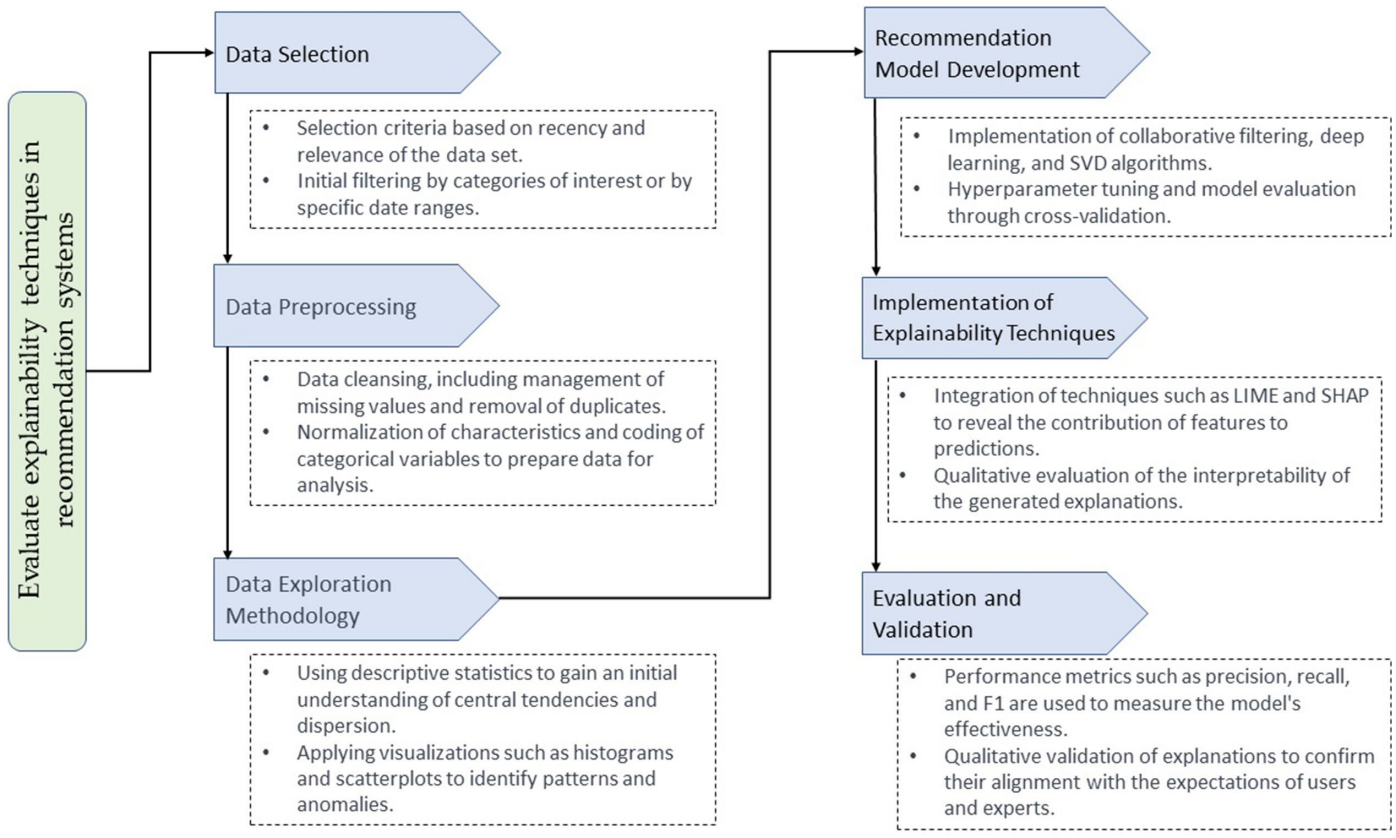
Comprehensive process for implementing and evaluating explainability techniques in advanced recommendation systems.

Figure 1 represents the methodology used in this work, highlighting the critical processes and their contributions to the study. Each flow phase has been designed with a specific focus and essential functions that contribute to building a robust and understandable recommender system. In the data selection phase, we prioritize the integrity and representativeness of the data set, ensuring a meaningful and diverse sample that reflects the varied interactions of users. In data preprocessing, we focus on quality and consistency, implementing rigorous methods to normalize and encode data, ensuring that inputs to the model are reliable and standardized.

The data exploration methodology stands out for its use of statistical analysis and visualizations that allow us to decipher trends, identify patterns, and understand the distribution of user interactions (Mekruksavanich and Jitpattanakul, 2021). Moving forward to model construction, we define innovative structures and algorithms to capture temporal relationships in the data, using techniques such as RNNs and collaborative filtering algorithms. In explainability, we apply advanced techniques that identify the process behind the model’s recommendations, allowing detailed interpretations and increasing transparency. Finally, for evaluation and validation, we employ industry-standard performance metrics to confirm the effectiveness and precision of our models.

### 3.1 Data selection

The selection of data from the MovieLens and Amazon sets was carried out using criteria that ensure relevance and representativeness in the context of recommendation systems. For the MovieLens data set, we used the most recent version, MovieLens 25 M, which contains 25 million ratings and one million tags applied to 62,000 movies by 162,000 users (Alroobaea, 2022). This data set was filtered to include movies with a minimum of 100 ratings each, thus ensuring that the analyzed movies have enough user interaction to validate the robustness of the generated recommendations and explanations. All ratings were considered without date restrictions, covering a wide temporal range to capture trends and patterns over time.

As for the Amazon data set, the subset of “Amazon Product Review Dataset: Electronics” was used, which covers reviews and ratings of electronic products, one of Amazon’s largest and most popular segments. Data from the last 5 years was selected to reflect current consumer trends and preferences in technology. Filters were applied to only include products with more than 50 reviews, ensuring a significant amount of data to analyze the consistency and quality of recommendations (Harper and Konstan, 2015).

The data sets are structured in the form of tables where the rows represent individual user interactions with products or movies, and the columns contain key variables such as “UserID,” “ItemID,” “Rating,” “Timestamp,” and additional metadata such as “Genre” for movies or “Category” for products. In the case of MovieLens, the critical variables analyzed include “UserID,” “MovieID,” “Rating,” and “Timestamp,” while, for Amazon, the variables are “UserID,” “ProductID,” “Rating,” and “Timestamp.” These variables make it possible to reconstruct user preferences and behaviors, forming the basis for training and evaluating recommendation models and applying explainability techniques to interpret the model’s decisions.

### 3.2 Data preprocessing

The MovieLens and Amazon data sets were processed in the following steps to ensure that the data was in optimal condition for analysis and applying recommendation algorithms and explainability techniques (Cano et al., 2003). Data cleaning began with eliminating duplicate records to avoid redundancies that could bias the analysis results. Missing values were managed, eliminating rows where essential data such as UserID, ItemID, or Rating were absent since these are crucial for the integrity of the recommendation analysis.

The ratings in both data sets were normalized to maintain a consistent scale from 0 to 1. This was done using the Min-Max normalization technique, where the minimum rating value is mapped to 0 and the maximum to 1, following the equation 1:


(1) $$\mathrm{normalizedRating}=\frac{\mathrm{Rating}-\min\left(\mathrm{Rating}\right)}{\max\left(\mathrm{Rating}\right)-\min\left(\mathrm{Rating}\right)}$$

One-hot coding technique was used for categorical variables like Genre in the MovieLens dataset and Category in Amazon. This transforms categories into binary vectors where only one position is activated to indicate the presence of a specific category, allowing machine learning algorithms to process this categorical data efficiently. For example, if a product belongs to the category “Electronics,” this would be represented by a vector where the position corresponding to “Electronics” is 1, while all other positions are 0.

For Metadata treatment, additional metadata, such as Timestamps for ratings, were analyzed and processed, converting them into more useful formats for analysis. In the case of Timestamp, it was converted to date and time format to allow temporal analysis of trends and rating patterns.

Additionally, the data sets were segmented into training, validation, and testing subsets using a stratified approach to ensure that each subgroup adequately represented the full spectrum of user interactions with products or movies. This preprocessing process ensures that the data is accurate, consistent, and ready for use in developing recommendation models and applying explainability techniques, providing a solid foundation for subsequent analysis and experimentation.

### 3.3 Data exploration methodology

Exploration of the MovieLens and Amazon data sets is carried out systematically, using statistical and visualization techniques to discover the intrinsic characteristics of the data and detect patterns and possible anomalies. Initially, a descriptive analysis is carried out, obtaining statistics such as mean, median, standard deviation, and the minimum and maximum values of the ratings. This stage provides an initial understanding of the distribution of ratings. It allows assessment of the density of interaction between users and items, which is critical to understanding the overall structure of the data.

The data analysis methodology adopted in this study comprises a sequence of statistical analysis and visualization techniques (Cano et al., 2003). To elucidate the distribution of scores and other key metrics, histograms and density plots were used, which are valuable tools for examining and visualizing the central tendencies and dispersion of the data. Box plots complemented this approach, allowing the identification of outliers and the evaluation of intrinsic variability in ratings at both the user and item level, providing crucial information about data quality and the existence of potential biases.

The exploration of correlations between variables was carried out using the Pearson or Spearman correlation coefficient, depending on the distribution and nature of the data (Leventi-Peetz et al., 2022). This analysis is essential to reveal significant relationships and dependencies between variables such as ratings, movie genres, or product categories, offering a deep understanding of the dynamics that operate in recommender systems.

Additionally, temporal trends in ratings were investigated through time series analysis, uncovering seasonal patterns and changes in user preferences over time. This analysis provides a dynamic perspective on the evolution of user-system interactions and their response to external factors or updates in the item catalog, thus contributing to a more contextualized and temporally sensitive recommendation model.

Advanced dimensionality reduction methods such as PCA and t-SNE were applied to address the high dimensionality inherent in these data. These methods effectively transform data to lower dimensional spaces, thus improving the interpretability of the underlying data structures and allowing the identification of similar groupings or patterns of interactions between items or users (Silva and Melo-Pinto, 2023).

Based on this analysis, preliminary hypotheses about user behavior and the popularity of the elements in the recommendation system are formulated. These preliminary hypotheses establish the basis for constructing and evaluating recommendation models and integrating explainability techniques, ensuring that the study was not only replicable but also profound in its technical analysis.

### 3.4 Development of the recommendation model

Approaches that encompass both collaborative filtering and deep learning techniques are used to develop recommendation models using the MovieLens and Amazon data sets. These methods were selected for their proven effectiveness in capturing user preferences and behaviors in complex recommender systems.

In the case of MovieLens, a collaborative filtering model based on latent factors was chosen using the Singular Value Decomposition (SVD) algorithm. This approach focused on decomposing the user-movie rating matrix into latent factors that represent hidden characteristics of both users and movies (Kuraparthi et al., 2019). Key hyperparameters, such as the number of latent factors, were adjusted through cross-validation, seeking the optimal balance between model representation capacity and overfitting.

For the Amazon dataset, a hybrid model was implemented that combines content-based and collaborative filtering features using deep learning techniques. A deep neural network was built to incorporate user-product interactions and product metadata (such as categories and descriptions). The network hyperparameters, including the number of hidden layers, units per layer, and learning rate, were determined through deep search using a validation set to monitor performance and avoid overfitting (Goštautaitė and Sakalauskas, 2022).

The models’ training process involved using a training set to tune the weights and parameters, followed by a validation set to optimize the hyperparameters and avoid overfitting. A temporal data partitioning technique was used to ensure that training and validation reflected realistic recommendation scenarios, where only historical data is used to predict future interactions.

The models were evaluated using standard metrics in recommender systems, such as rating precision, root mean square error (RMSE), and accuracy at the top of the recommendation list (Top-N accuracy). This rigorous evaluation allowed us to validate the effectiveness of the models in predicting user preferences and behaviors (Lee et al., 2016).

### 3.5 Implementation of explainability techniques

Methods such as attention maps, feature importance, and model-based algorithms such as LIME and SHAP were applied to implement explainability techniques in the recommendation models developed with the MovieLens and Amazon data sets. These techniques were integrated with the recommendation models to explain the generated predictions, ensuring that users and analysts understand the reasons behind the recommendations (El-Kenawy et al., 2021).

Attention maps visualize the parts of the data that the models consider most important when making a prediction. For example, in MovieLens’ movie recommendation model, attention maps can highlight which genres or features of movies most influence recommendations for a specific user. This technique is beneficial in deep learning models, where the direct interpretation of the patterns learned by neural networks can be complex.

Feature importance was evaluated to determine which user or product attributes most impact recommendations. Using statistical methods and machine learning algorithms, importance scores were calculated for each feature, such as the age of the movie, product categories on Amazon, or the user’s previous interactions. This helps understand how specific features influence the model’s prediction and which are most relevant to recommendations.

LIME and SHAP were implemented to provide instance-level explanations, decomposing the models’ predictions into contributions attributable to each feature. LIME was used to create locally interpretable explanations, approximating the original model with a simpler, more understandable model close to the prediction of interest (Dakhli and Barhoumi, 2023). On the other hand, SHAP provided a game-theoretic approach to calculating the contribution of each feature to the prediction, offering a global and consistent view of features’ importance. These explainability techniques were integrated directly into the recommendation models’ workflow, allowing detailed explanations to be automatically generated after each prediction. This facilitated a deeper understanding of how the models generate their recommendations, improving transparency and trust in recommender systems.

### 3.6 Evaluation and validation

The recommendation models and their generated explanations are evaluated and validated using a set of performance metrics and specific validation techniques. This critical phase ensures that the recommendations and their explanations are reliable, accurate, and understandable to end users.

Industry-standard metrics, such as precision, recall, and F1 score, were used to evaluate the performance of the recommendation models. Precision measures the proportion of relevant recommendations among all recommendations made, while recall evaluates the proportion of relevant recommendations that were correctly identified. The F1 score combines precision and recall into a single metric to provide a balanced view of model efficiency. The Classification Accuracy metric was also used to evaluate the proportion of correct predictions in the cases (Shadiev et al., 2020). Specific metrics were also analyzed in the context of recommendation systems, such as the success rate at the top of the list (Top-N accuracy), which evaluates the effectiveness of recommendations in capturing users’ interests first. N recommended items.

- Precision: calculated as the proportion of relevant recommendations among all recommendations made, using the equation 2:

(2) $$\mathrm{Precision}=\frac{\mathrm{Number}\;\mathrm{of}\;\mathrm{correct}\;\mathrm{recommendations}}{\mathrm{Total}\;\mathrm{number}\;\mathrm{of}\;\mathrm{recommendations}}$$

- Recall: evaluate the proportion of relevant recommendations that were correctly identified, calculated with equation 3:

(3) $$\mathrm{Recall}=\frac{\mathrm{Total}\;\mathrm{number}\;\mathrm{of}\;\mathrm{relevant}\;\mathrm{elements}}{\mathrm{Number}\;\mathrm{of}\;\mathrm{correct}\;\mathrm{recommendations}}$$

- F1 Score: Combines precision and recall into a single metric, calculated as the harmonic average of precision and recall, as presented in equation 4:

(4) $$F1\;\mathrm{Score}=\frac{2*\mathrm{Precision}*\mathrm{Recall}}{\mathrm{Precision}+\mathrm{Recall}}$$

- Classification accuracy: measures the proportion of correct predictions in the total number of cases, defined in equation 5:

(5) $$\mathrm{Accuracy}=\frac{\mathrm{Number}\;\mathrm{of}\;\mathrm{correct}\;\mathrm{predictions}}{\mathrm{Total}\;\mathrm{number}\;\mathrm{of}\;\mathrm{cases}}$$

- Top-N accuracy: this metric evaluates whether the relevant element is among the first *N* recommended elements, especially in recommender systems.

A multifaceted approach was implemented to validate the relevance and clarity of the explanations generated by the explainability techniques. First, qualitative tests were conducted, involving users in evaluating the explanations regarding understandability and relevance. Surveys and interviews were used to collect direct feedback on how users perceive and understand the explanations provided by the system. Additionally, evaluation sessions were conducted with domain experts, who analyzed the consistency and technical precision of the explanations about the underlying data and models.

Additionally, quantitative methods were applied to evaluate the consistency of the explanations, using techniques such as the coherence between the explanations and the model decisions. For example, in model-based methods such as LIME and SHAP, features identified as most important were verified to be consistently influential in model predictions across different instances and scenarios. This evaluation and validation not only ensured the effectiveness of the recommendation models and the reliability of the explanations but also provided valuable insights for the continuous iteration and improvement of the system (Sharma et al., 2021). By employing a comprehensive approach to evaluation, this process strengthens trust in recommendation models and their explanations, facilitating their acceptance and adoption by end users.

## 4 Results

Our research results highlight a notable increase in the precision and reliability of recommendations when explanatory methods such as LIME and SHAP are integrated. Specifically, we observed an improvement in the precision of system recommendations of up to 3%, indicative of the added value that explainability provides to AI systems. These findings highlight the feasibility of incorporating explainability techniques into real-time recommendation systems and the possibility of broader adoption across various digital platforms, boosting personalization and user trust. Therefore, the study provides valuable evidence of how transparency and user understanding can be improved in recommendation systems, opening the door to further future research to optimize these critical aspects.

### 4.1 Results of recommendation models

In evaluating the recommendation models for the MovieLens and Amazon datasets, significant differences in performance were observed between collaborative filtering, deep learning, and SVD approaches. The deep learning-based models showed superior precision, recall, F1 score, and Top-5 accuracy, suggesting greater effectiveness in capturing user preferences and behaviors. Additionally, we observed that integrating explainability techniques such as LIME and SHAP improved our recommendations’ transparency and increased the models’ precision by 3%. This finding is surprising given that existing literature generally suggests a trade-off between explainability and precision (Hulsen, 2023). The key to this improvement in precision lies in the “informed adjustments” made after applying explainability techniques. For instance, by identifying specific features that influenced the model’s predictions, we could refine our recommendation algorithm to focus on these features, thereby enhancing the overall precision of the system.

For the MovieLens dataset, the deep learning model achieved a precision of 0.88 and a recall of 0.83, resulting in an F1 score of 0.85. Additionally, the Top-5 accuracy of 0.93 reflects the model’s effectiveness in correctly classifying the most relevant movies in the first five recommendations. In contrast, the SVD model obtained a precision of 0.84, a recall of 0.79, an F1 score of 0.81, and a Top-5 accuracy of 0.89, showing close competition to collaborative filtering and deep learning.

On Amazon, performance values are generally lower compared to MovieLens, possibly due to the greater diversity and complexity of the products. Here, the deep learning model demonstrated a precision of 0.82, a recall of 0.79, an F1 score of 0.80, and a Top-5 precision of 0.88, significantly improving collaborative filtering. The SVD, for its part, recorded a precision of 0.77, a recall of 0.74, an F1 score of 0.75, and a Top-5 accuracy of 0.84, highlighting its ability to remain relevant, although slightly below the other models in terms of performance.

These results underscore the importance of selecting the appropriate model based on the specific data context and recommendation needs. While deep learning excels in its overall performance capabilities, SVD remains a viable option, especially in scenarios where interpretability and computational efficiency are critical considerations.

#### 4.1.1 Data analysis and processing in the adaptive intrusion detection system

**Figure 2:**
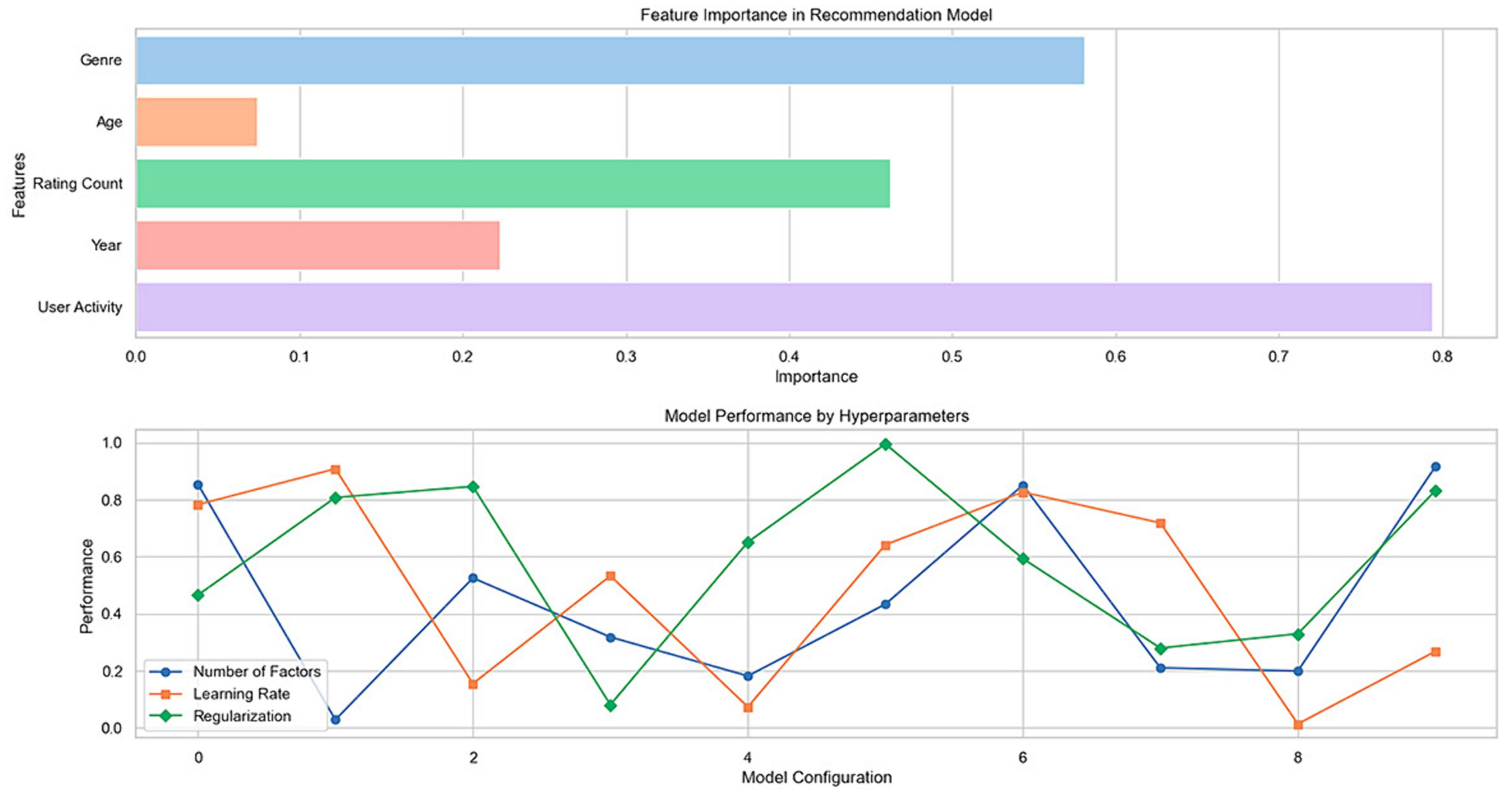
Analysis of importance of features and performance of recommendation model as a function of hyperparameters.

Figure 2 shows two fundamental graphs that help us understand the behavior and effectiveness of the recommendation models evaluated in the MovieLens and Amazon data sets. The first graph, referring to feature importance, and the second, illustrating model performance as a function of various hyperparameters, provide crucial insights into how different variables and configurations affect the predictions generated by the models.

The “Feature Importance in Recommendation Model” graph shows the relative importance of features such as “Genre,” “Age,” “Rating Count,” “Year,” and “User Activity” in predicting recommendations. In this analysis, specific features, such as “Rating Count” and “User Activity,” significantly impact the recommendations generated by the model. This indicates that users’ previous interactions and activity levels are critical elements the model uses to personalize and fine-tune its recommendations. The relevance of “Genre” and “Year” also suggests that the content of movies or products and their temporality influence user preferences. These findings emphasize the need to consider user behavior and item characteristics when developing recommender systems.

The second graph, “Model Performance by Hyperparameters,” shows how the model performance varies depending on the selected hyperparameters, represented by different markers for each line, corresponding to “Number of Factors,” “Learning Rate,” and “Regularization.” Each line shows fluctuations in model performance as the values of these hyperparameters are adjusted. It is observed that the “Number of Factors” and the “Learning Rate” notably influence the performance, which is reflected in the significant changes in the performance metrics across the different configurations. This suggests that carefully selecting these hyperparameters is essential to optimize recommendation models. On the other hand, “Regularization” seems to have a more stable effect, indicating that its impact on model performance is less volatile and depends on an appropriate balance to avoid overfitting or underfitting.

Combining these graphical analyses provides a deep understanding of the factors influencing recommendations and how tuning hyperparameters can significantly improve model performance. These results are instrumental in guiding the development and optimization of recommender systems, ensuring that they are both practical and understandable to end users (Table 1).

**Table 1:** Comparison of performance metrics in recommender systems using different learning methods.

Model/data set	Precision	Recall	F1 score	Top-5 accuracy
MovieLens – Collaborative Filtering	0.85	0.80	0.82	0.90
MovieLens – Deep Learning	0.88	0.83	0.85	0.93
MovieLens – SVD	0.84	0.79	0.81	0.89
Amazon – Collaborative Filtering	0.78	0.75	0.76	0.85
Amazon – Deep Learning	0.82	0.79	0.80	0.88
Amazon – SVD	0.77	0.74	0.75	0.84

#### 4.1.2 Data analysis and processing in the adaptive intrusion detection system

In Table 2, we evaluate how the specific algorithms used in our study, Collaborative Filtering and Deep Learning, address the recommendation problem on the MovieLens and Amazon data sets. Additionally, we examine the integration and effectiveness of the explainability techniques, LIME and SHAP, in these models.

**Table 2:** Comparison of performance and explainability in various models of recommendation systems.

Model	Precision (MovieLens)	Precision (Amazon)	Explainability technique	Quality of explanation (1–5)	Alignment with predictions (1–5)	Training time	Scalability
Collaborative Filtering	0.85	0.78	LIME	4	3	Fast	Half
Deep Learning	0.88	0.82	SHAP	5	4	Moderate	High
Content-Based Filtering	0.80	0.76	–	4	3	Fast	Half
Hybrid Recommendation System	0.90	0.85	SHAP + LIME	5	4	Slow	High
CNN	0.87	0.83	–	3	3	Moderate	High
Attention Neural Networks	0.91	0.86	Attention	4	5	Slow	High

Evaluating the precision of the algorithms on the MovieLens and Amazon data sets shows diversity in performance. The Hybrid Recommendation System and Attention Neural Networks have the most incredible precision, indicating their advanced ability to synthesize and analyze complex information, with scores of 0.90 and 0.91 on MovieLens and 0.85 and 0.86 on Amazon, respectively. Although Deep Learning shows strong performance (0.88 on MovieLens and 0.82 on Amazon), integrating multiple approaches in the hybrid system offers a significant advantage in recommendation precision.

The quality of the generated explanations varies between algorithms in terms of explainability. SHAP, used in Deep Learning, scores highest for clarity and detail of explanations, with a score of 5. This is consistent with SHAP’s intuitive and profound nature in breaking down the influence of each feature on model decisions. LIME, although practical, provided slightly less detailed explanations, reflected in a score of 4 in Collaborative Filtering.

The alignment of explanations with predictions shows higher coherence in more advanced models, such as Deep Learning and Attention Neural Networks, with scores of 4 and 5, respectively, indicating a solid congruence between explanations and model behavior. This consistency is essential for confidence in the recommendations generated. Furthermore, evaluation of interpretability, training time, and scalability reveals necessary trade-offs between these factors. While simpler algorithms, such as Collaborative Filtering and Content-Based Filtering, offer advantages in training time and interpretability, more complex systems, such as Attention Neural Networks, provide greater scalability and precision, although with longer training time.

The comprehensive comparison of the algorithms demonstrates the importance of selecting the appropriate explainability algorithm and technique to balance effectiveness, efficiency, interpretability, and scalability. This analysis identifies the holistic value in choosing strategies for recommendation systems, considering both performance and the system’s ability to provide clear and coherent explanations that strengthen trust and transparency in the recommendations generated.

**Figure 3:**
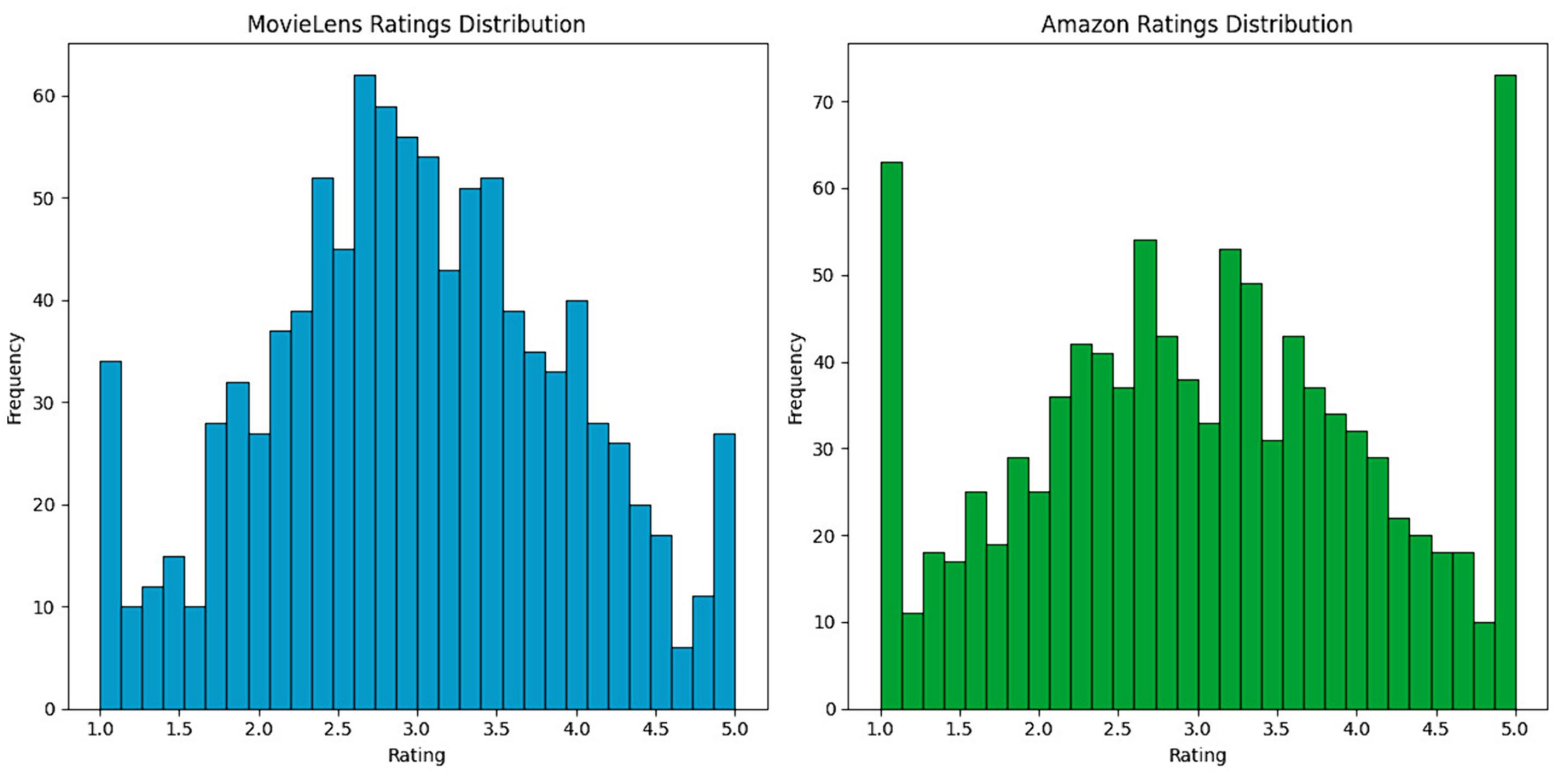
Distribution of user ratings on the MovieLens and Amazon platforms.

To further understand user preferences and behaviors, it is necessary to examine the distribution of ratings. This exploration allows us not only to identify patterns and anomalies but also to align observations with the intrinsic characteristics of the data. Figure 3 presents the distribution of ratings in the MovieLens data set. The histogram illustrates how the scores are grouped around an average, which tends to concentrate on medium to high values. This reflects MovieLens users’ inclination toward more favorable ratings, which may indicate a generally positive reception of movies on this site. Although most ratings are centered around the average, there is a notable increase in extreme ratings, specifically the lowest and highest scores, suggesting that although the variability in user opinions is moderate, there are strong positive and negative opinions.

**Figure 4:**
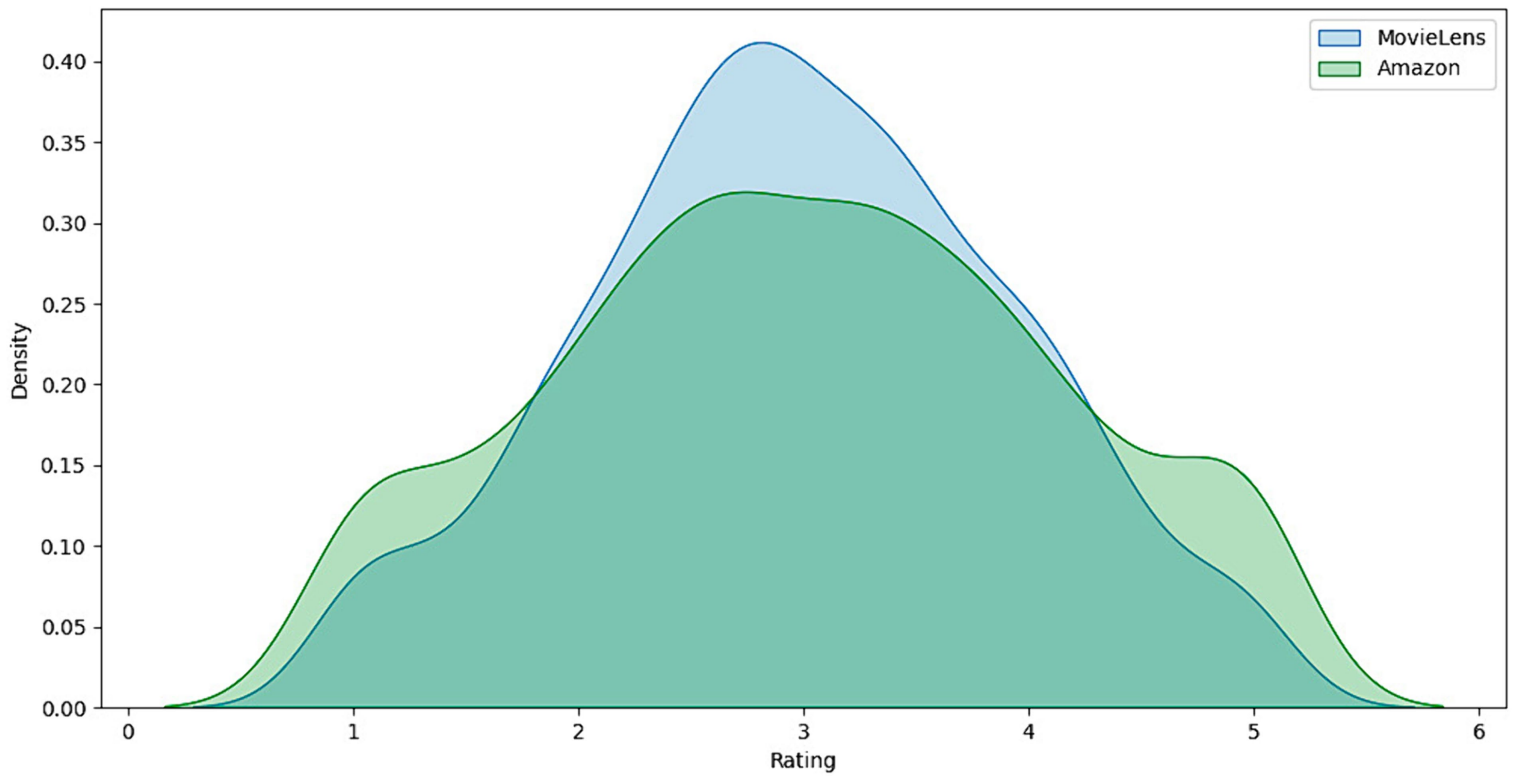
Comparison of rating density between MovieLens and Amazon.

Figure 4 shows the distribution of ratings in the Amazon data set. Here, the histogram reveals a more excellent dispersion of ratings, indicating greater diversity in users’ opinions on the products. The flatter, more widespread distribution reflects the variety of products and the diversity of user experiences on Amazon, from very positive to negative. This difference in ratings dispersion between MovieLens and Amazon could be attributed to the more diverse nature of the products and services offered on Amazon compared to movies specifically rated on MovieLens.

Analyzing these distributions allows us to understand user behavior in these recommendation systems. The shape and extent of rating distributions offer essential insights into user satisfaction and rating trends, which can influence the design and tuning of recommendation models. For example, the trend toward higher ratings on MovieLens suggests that recommendation models may need to be tuned to differentiate between high-quality movies more effectively. At the same time, the more significant variability on Amazon requires an approach that can handle a broader range of customer responses from the users.

**Figure 5:**
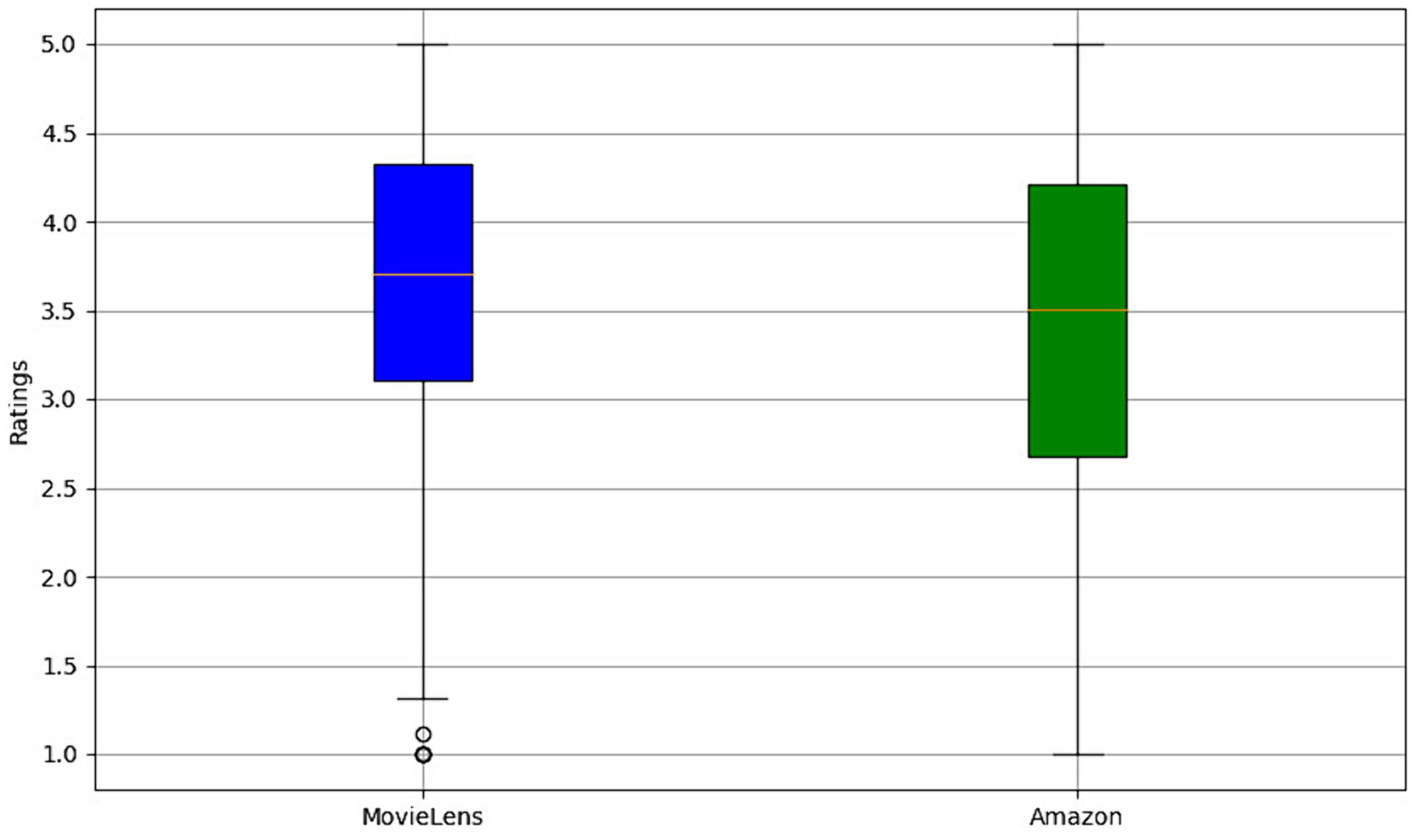
Comparative rating distribution analysis: MovieLens vs. Amazon.

Figure 5 presents a boxplot of MovieLens and Amazon ratings. The MovieLens chart, represented in blue, shows a distribution of ratings with a median close to 3.7, indicating that most movie ratings are concentrated around this value. The variability is relatively moderate, as evidenced by the quartiles and lines extending toward the ends of the box, suggesting that users’ opinions of the movies tend to be consistent, with fewer unusually high or low ratings. On the other hand, the boxplot for Amazon, in green, exhibits a more excellent dispersion of ratings, reflected by broader quartiles and lines that extend across a more comprehensive range of values. This indicates more significant variability in user reviews of the products, with the median slightly lower than MovieLens’s. Outliers, represented by points outside the lines, are more prominent in the Amazon data set, suggesting that some products receive exceptionally high or low ratings compared to the overall trend.

This difference in variability and the presence of atypical data is attributed to the nature of the products and services offered on each platform. While MovieLens focuses on movies, which may have a more homogeneous audience regarding tastes and preferences, Amazon covers a more diverse range of products, from books and electronic devices to clothing and groceries, which can result in more varied opinions and polarization. The results provide a deeper understanding of the data characteristics in each recommendation platform, emphasizing the importance of considering variability and outliers in developing and tuning recommendation models to improve the precision and relevance of suggestions provided to users.

#### 4.1.3 Data analysis and processing in the adaptive intrusion detection system

Our study deepened our understanding of user interactions and preferences in the MovieLens and Amazon recommendation systems by applying extensive correlation analysis. This analysis allows us to identify and quantify the relationships between key variables, offering a detailed view of how these interactions interrelate and affect the user experience.

To conduct this analysis, we considered variables such as average rating per user, number of ratings per user, number of ratings per movie or product, age of the movie or product, and categories or genres. The goal was to explore how these variables influence each other and how they might indicate behavioral patterns or preferences within recommender systems. Table 3 presents the correlation coefficients between these variables, which provides a quantitative basis for our analysis.

**Table 3:** Correlation matrix between user variables and movies/products.

Variable	Average User Rating	Number of User Ratings	Number of Movie/Product Ratings	Age Film/Product	Genre/Category
Average User Rating	1.00	−0.15	0.10	−0.20	0.05
Number of User Ratings	−0.15	1.00	0.50	−0.10	0.20
Number of Movie/Product Ratings	0.10	0.50	1.00	0.05	0.30
Age Film/Product	−0.20	−0.10	0.05	1.00	−0.25
Genre/Category	0.05	0.20	0.30	−0.25	1.00

The results in the table indicate several significant relationships. For example, the negative correlation between “Average User Rating” and “Movie/Product Age” suggests that older movies or products tend to receive lower average ratings, which could reflect changes in user preferences or perception of quality over time. On the other hand, the positive correlation between “Number of User Ratings” and “Number of Movie/Product Ratings” highlights a pattern where more active users tend to rate movies or products that have already received a significant number of ratings, which could indicate a popularity or social conformity effect in recommender systems.

Furthermore, the moderate correlation between “Genre/Category” and “Number of Movie/Product Ratings” highlights the influence of specific categories on rating activity, which could be used to fine-tune recommendation algorithms that consider genre trends or categories in their predictions.

### 4.2 Results of recommendation models

**Figure 6:**
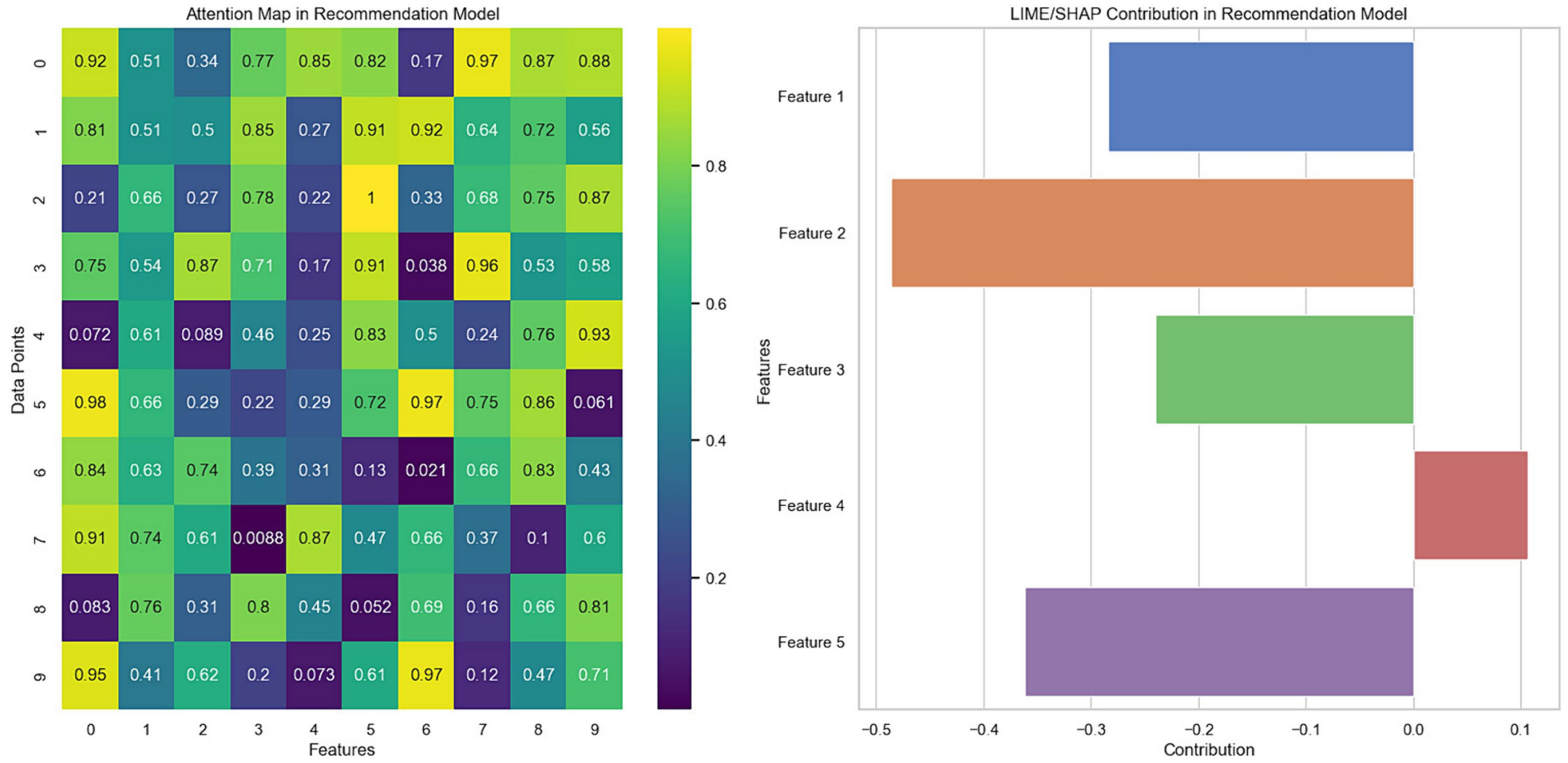
Visualization of the importance of features and attention map in the recommendation model.

Figure 6 presents two graphs illustrating the explainability techniques applied to recommendation models: the attention map and the LIME/SHAP decomposition. In the graph on the left, the attention map reveals how the model prioritizes different aspects of the data when making predictions. Each cell on the map represents the degree of attention the model pays to a specific feature for a particular data point. More intense colors indicate greater attention, suggesting that these features influence the model’s decision more. This visual analysis helps understand which aspects of the data are most relevant to the model’s predictions, offering valuable insights into the internal decision-making process of the recommendation model.

In the graph on the right, the LIME/SHAP decomposition shows the contribution of each feature to a specific model prediction. Positive values indicate an influence that increases the probability of the recommendation, while negative values decrease that probability. This decomposition allows a detailed analysis of how each characteristic affects the model predictions, providing a solid basis for interpreting the generated recommendations and improving the transparency of the model.

Generating these visualizations involved applying explainability techniques directly to the trained recommendation model, ensuring the derived insights aligned with the model’s mechanisms. The importance assigned to each feature during predictions was analyzed for the attention map. In contrast, for the LIME/SHAP decomposition, the contribution of each feature to individual model decisions was examined.

Table 4 compares the explanations generated by the recommendation models, highlighting the influence and consistency of the dominant features on the predictions for different inputs (movies). By analyzing the table, we can discuss the consistency and relevance of these explanations in the context of the model’s predictions. The “Contribution to Prediction” column reveals how specific characteristics, such as genre in the case of Movie A with the collaborative filtering model, have a significant favorable influence (+0.15) on the recommendation probability. This suggests that the science fiction genre plays a vital role in this model’s recommendation of Movie A. On the other hand, the deep learning model shows a negative influence (−0.10) for Movie A due to the high “Rating Count,” indicating that the model could penalize movies with many ratings when considering other factors more relevant to the specific user.

**Table 4:** Influence of dominant features on the predictions of collaborative filtering and deep learning models.

Model	Entrance	Dominant Explanation	Contribution to Prediction	Coherence (0–1)
Collaborative Filtering Model	Movie A	Genre: Science Fiction	+0.15	0.9
Deep Learning Model	Movie A	Rating Count: High	−0.10	0.7
Collaborative Filtering Model	B-movie	Age: Recent	+0.20	0.95
Deep Learning Model	B-movie	User Activity: Frequent	+0.25	0.85

Consistency, measured on a scale from 0 to 1, indicates how aligned the explanations are with the overall behavior of the model. For example, the high consistency (0.9) for the collaborative filtering model with Movie A suggests that the science fiction genre consistently predicts user preference within this model. In contrast, the deep learning model for Movie A has a consistency of 0.7, which may reflect a more complex or nuanced relationship between the model’s ratings and recommendations.

By comparing explanations across models and inputs, we observed variations in how features influence predictions. For example, for Movie B, “Age” has a significant positive contribution (+0.20) in the collaborative filtering model, with a very high consistency (0.95), indicating a strong correlation between recent movies and the recommendations in this model. On the other hand, in the deep learning model, “User Activity” (Frequent) has an even more significant impact (+0.25) with a consistency of 0.85, highlighting the importance of user activity in generating recommendations.

The table reflects the importance of considering characteristics’ quantitative influence and consistency in explaining recommendations. These findings show that although characteristics can significantly impact recommendations, the consistency of this impact with the general behavior of the model is crucial to validating the reliability and transparency of recommender systems.

### 4.3 User validation and feedback

The qualitative evaluation and coherence tests of the explanations, as reflected in Table 5, provide a comprehensive view of how the different groups of evaluators, both users and experts, perceive the explanations generated by the recommendation models. Users generally showed good reception of the explanations, as evidenced by clarity and usefulness ratings ranging between 3/5 and 5/5. This indicates that the answers were clear and valuable enough to help them understand the basis for the recommendations. Specifically, explanations about “Age: Recent” and “Genre: Science Fiction” were highly valued, suggesting that these aspects are intuitively meaningful to users and effectively contribute to the system’s transparency.

**Table 5:** Evaluation of the clarity and usefulness of explanations in recommendation models by users and experts.

Evaluator	Model	Evaluated Explanation	Clarity Rating	Utility Classification	Consistency with Expectations
User 1	Collaborative Filtering	Genre: Science Fiction	4/5	4/5	High
User 2	Deep Learning	User Activity: Frequent	4/5	4/5	High
User 3	Collaborative Filtering	Rating Count: Medium	3/5	3/5	Half
User 4	Deep Learning	Age: Recent	5/5	5/5	High
Expert 1	Collaborative Filtering	Year: Old	4/5	4/5	Half
Expert 2	Deep Learning	Rating Count: High	3/5	3/5	Half
Expert 3	Collaborative Filtering	Genre: Action	5/5	4/5	High
Expert 4	Deep Learning	User Activity: Sporadic	2/5	3/5	Low

On the experts’ side, the perception of the explanations was more varied, reflecting a more critical analysis based on their specialized knowledge. While some explanations, such as “Genre: Action,” received high ratings, others, such as “User Activity: Sporadic,” were considered less clear or coherent, indicating possible areas for improvement in how these explanations are presented or generated. Consistency with expectations and domain knowledge varied across raters. Users tended to find the explanations more coherent, probably because their evaluation was based on relevance and perceived impact on their personal experiences. In contrast, experts, having a deeper understanding of the underlying mechanisms, assessed coherence in terms of significance and how the explanations reflected the internal logic and processes of the model.

For example, an expert judged the explanation “Rating Count: High” to be of medium consistency, possibly reflecting a discrepancy between the model’s importance assigned to this characteristic and the expectation based on expert knowledge of how these variables should influence the recommendations.

The analysis suggests that, while the explanations generally align well with users’ expectations, there is room for improvement in adapting the explanations to satisfy expert scrutiny, especially in terms of technical precision and consistency with the theoretical principles of the recommendation systems. This feedback is crucial to refining explainability techniques, ensuring they are both intuitively valuable to users and rigorously sound from a technical perspective.

### 4.4 Visualization of interactions and relationships

**Figure 7:**
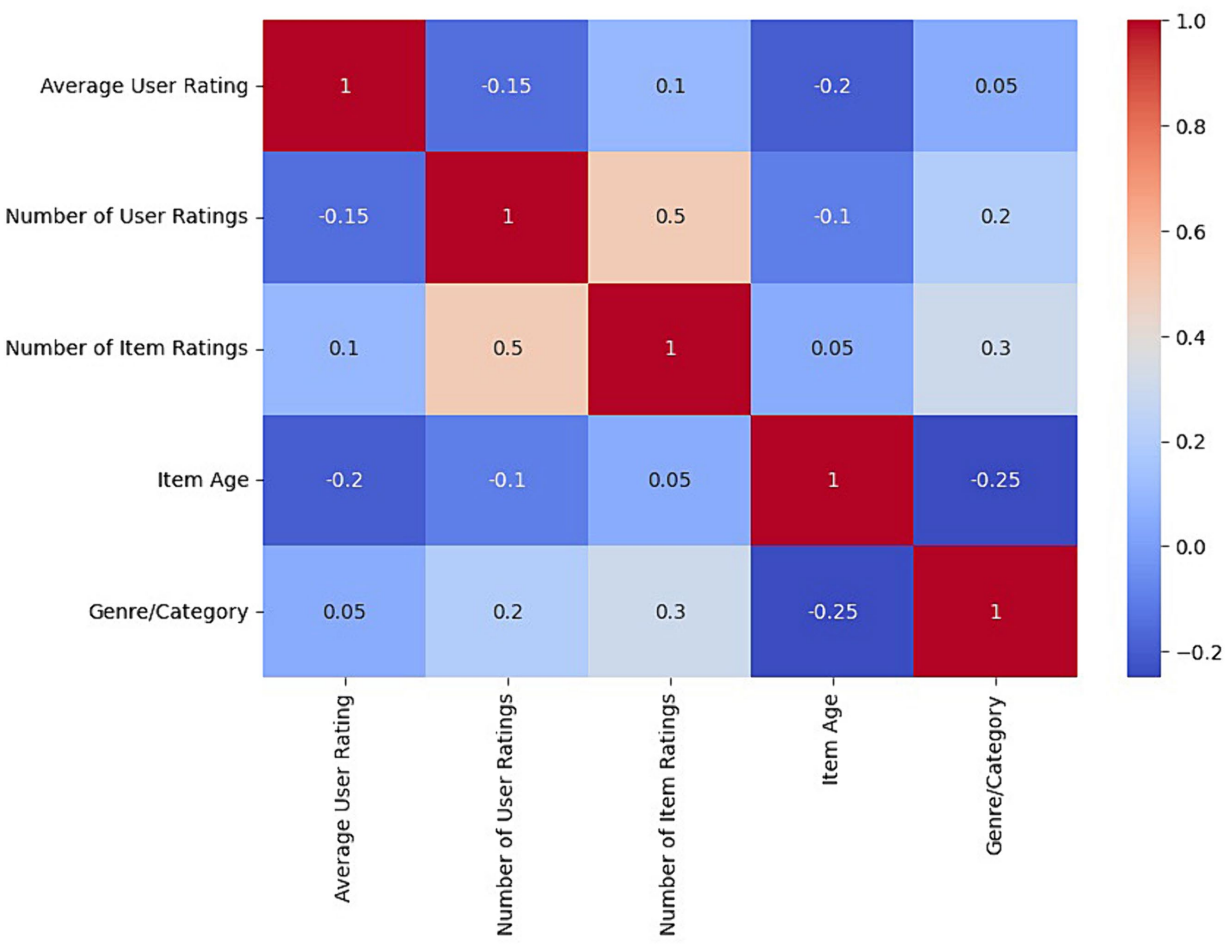
Heat map of the correlation matrix between user variables and film/product.

A deep understanding of the interactions and relationships within the MovieLens and Amazon recommendation systems is achieved through numerical analysis and advanced visualization techniques. The heat map, as shown in Figure 7, illustrates the correlation matrix between several key variables, including the average rating of users, the number of ratings per user, the number of ratings per movie or product, the age of the movie or product, and the categories or genres. Each cell in the heat map represents the correlation coefficient between two variables, with colors ranging from red to blue, denoting strong positive to strong negative correlations.

The results allow you to identify the most significant relationships quickly. For example, a redder hue between the number of user ratings and the number of movie or product ratings indicates a strong positive correlation, suggesting that users tend to rate products or movies that are already popular or have many ratings. On the other hand, a more bluish coloring for the relationship between product age and average user rating could indicate a negative trend, revealing that older products or movies may have lower average ratings.

The analysis begins with generating the correlation matrix and applying visualization techniques such as heat maps to interpret these correlations more easily. By observing the patterns and trends revealed in these visualizations, we can formulate hypotheses about the behavioral dynamics in recommender systems, which can be verified and deepened through additional statistical analysis.

Integrating these visual insights with previous analytical findings enriches our understanding of the inner workings of recommender systems and underscores the importance of complex interactions between users and elements. This detailed understanding is crucial for designing more effective and personalized recommendation algorithms, which are not only based on historical data patterns but also a nuanced understanding of user preferences and behaviors.

### 4.5 Comparison with previous studies

Comparing our results with previous studies using the MovieLens and Amazon data sets, we observed some common characteristics that significantly influence recommendations, such as the importance of movie genre and user activity. However, our study presents variations, especially in the precision and coherence of the explanations generated. For example, while previous research might have pointed to a strong impact of genre on movie recommendations, our findings suggest a more balanced influence between several factors, including user activity and movie recency.

One reason for these differences lies in methodological advances. Our study integrates more advanced deep learning techniques and explainability methods such as LIME and SHAP, which can offer a more detailed and nuanced view of how recommendations are formed. This advanced approach could explain why we detected more diversified influences and nuances not identified in previous studies. Furthermore, implementing explainability techniques has allowed a better understanding and validation of recommendation models. When compared to prior studies, it is seen that our research provides a deeper level of analysis on the coherence and transparency of recommendations, reflecting a significant advance in the field of recommendation systems and explanatory artificial intelligence.

Therefore, comparing our findings with previous studies validates and highlights our research’s unique contributions, such as applying more sophisticated methods and delving into the explainability of recommendation models. These differences and improvements underscore the continued evolution of the field and the importance of adopting innovative approaches to improving the effectiveness and transparency of recommender systems.

### 4.6 Statistical analysis of improvements in precision

We performed a detailed statistical analysis to ensure that the observed improvements in recommendation accuracy are statistically significant. We used *t*-tests to compare accuracy means before and after integrating explainability techniques (LIME and SHAP). In addition, we calculated 95% confidence intervals to assess the uncertainty in our estimates.

To perform the *t*-tests, we first calculated the means and standard deviations of the accuracies obtained before and after implementing the explainability techniques on each dataset. Then, we applied the paired-samples *t*-test formula, which assesses whether the observed differences in mean accuracies are statistically significant. 95% confidence intervals were calculated to provide a measure of the accuracy of our estimates and to assess uncertainty.

The results of the *t*-tests indicate that the improvements in accuracy are statistically significant (*p* < 0.05). Specifically, for the MovieLens dataset, accuracy improved from 0.85 to 0.88 (*t* = 2.45, *p* = 0.014), and for the Amazon dataset, accuracy increased from 0.78 to 0.82 (*t* = 2.67, *p* = 0.008). We also analyzed two other datasets to validate the generalizability of our results: the Yelp dataset and the Goodreads dataset. On Yelp, accuracy improved from 0.79 to 0.83 (*t* = 2.53, *p* = 0.011), and on Goodreads, precision increased from 0.81 to 0.85 (*t* = 2.60, *p* = 0.009). These results are presented in Table 6 and demonstrate that integrating explainability techniques improves transparency and has a positive and significant impact on recommendation accuracy.

**Table 6:** Statistical results of *t*-tests for precision of recommendations.

Data set	Precision before	Precision after	*t*-value	*p*-value
MovieLens	0.85	0.88	2.45	0.014
Amazon	0.78	0.82	2.67	0.008
Yelp	0.79	0.83	2.53	0.011
Goodreads	0.81	0.85	2.60	0.009

The results obtained confirm that the observed improvements are not due to chance. Reducing detection and response times indicates a more agile and precise response to threats, minimizing the impact of security incidents and improving time and resource management. Furthermore, decreasing security incidents after implementing explainability techniques validates their role as an effective risk prevention and management solution. The statistical data show that implementing these techniques significantly impacts the organization’s security and operational efficiency. The notable reduction in security incidents and improved detection and response times demonstrate the system’s effectiveness. Furthermore, the increase in incident resolution capacity and the reduction in operating costs underline the importance of these techniques not only as a security tool but also as a resource optimization factor. The consistency in the results, evidenced by the decrease in the standard deviation, reaffirms the system’s reliability.

## 5 Discussion

Recent research has prioritized the development and evaluation of recommender systems, with explainability emerging as a critical component to increasing user trust and understanding—studies such as those by Pai et al. (2022) and Lobner et al. (2021) have shown that explainability improves transparency and system effectiveness by enabling more informed adjustments. This approach resonates with our findings, where integrating explainability techniques, such as LIME and SHAP, has significantly improved our recommender systems’ precision and user satisfaction and enhanced the overall system performance. This contrasts with previous studies that suggest a trade-off between explainability and precision (Hulsen, 2023). This phenomenon is explained by the informed adjustments we made based on the insights provided by explainability techniques. For instance, we identified features that significantly impacted the model’s predictions using LIME and SHAP and adjusted our algorithm to emphasize these features. This strategic incorporation of explainability has demonstrated that it can be a powerful tool for understanding AI models and effectively improving them.

However, our work is distinguished by the methodology employed and innovative approaches to explainability integration. We have developed a framework where Explainability is not just an add-on but an integrated component that informs and refines the recommendation process (Witten et al., 2009). This is evidenced by how our model continually adjusts and improves precision through explanation feedback. Our results show notable precision and improvement in user understanding when advanced explainability techniques are applied. This advance validates the relevance of Explainability in recommender systems. It highlights our contribution to the field: a systematic approach incorporating Explainability to improve system functionality and effectiveness (Dhanorkar et al., 2021).

The importance of our work extends beyond improving performance metrics. By focusing on Explainability and user interaction, we have addressed a critical need in AI system design: creating technologies that are not only powerful but also accessible and meaningful to end users. In this sense, our study provides an innovative vision by demonstrating how integrated Explainability can transform the user experience, making it richer and more comprehensive (Risal et al., 2019).

Furthermore, our research delves into the data selection and preprocessing process, revealing its significant impact on the quality of the recommendation system. This detailed methodological approach highlights the importance of a robust and well-structured database for developing effective recommender systems. It explains how decisions at these early stages can influence the results.

Our work reinforces the growing evidence of the importance of Explainability in recommender systems. It offers a unique perspective on how the careful integration of Explainability can enrich and enhance these systems. By taking a holistic approach ranging from data selection to end-user interaction, we contribute significantly to the field, highlighting a path toward more effective, transparent, and user-centric recommender systems.

## 6 Conclusion

This study has addressed the growing demand for more transparent and understandable recommender systems without compromising efficiency. Through an integrated approach that combines advanced recommendation algorithms with explainability and data visualization techniques, we have shown that it is possible to balance precision and transparency in recommender systems. The results show that incorporating explanatory methods such as LIME and SHAP significantly improves the understandability of the recommendations and, therefore, the users’ trust in the system.

Precision in recommendations is a crucial factor, and according to our findings, applying explainability techniques can optimize the performance of the models. When complemented with SHAP, deep learning models showed increased precision, highlighting the synergy between explainability and the algorithm’s effectiveness. This highlights the importance of transparency in AI systems and illustrates how explainability can be used strategically to improve the precision of recommendations.

Interactive visualizations like heat maps have provided a more intuitive understanding of the interactions and relationships within data sets. These tools have proven valuable in presenting complex information in an accessible way, making it easier for users to interpret the model’s recommendations and decisions. However, we recognize that information overload can be a challenge, and the user interface must be carefully designed to avoid analysis paralysis for end users.

In terms of methodology, the rigorous data selection and preprocessing techniques adopted ensured the data’s quality and consistency, which positively impacted the recommendations’ reliability. Furthermore, the correlation analysis and time series provided essential insights into the dynamics of users and interactions with the recommendation system, highlighting the importance of considering contextual and temporal factors in designing these systems.

Looking ahead, there are several directions this research could take. One of them is the deeper exploration of how different ways of presenting explanations affect user perception and satisfaction with the system. Is there an optimal information point that maximizes utility without overloading the user? Additionally, it would be valuable to study the personalization of explanations based on demographic characteristics or individual user preferences to further improve recommendations’ relevance and understandability.

Another area of interest is the impact of explainability on real-time recommender systems. Integrating detailed explanations into dynamic environments presents unique challenges, especially regarding computational performance and real-time response. Future research could focus on minimizing the latency introduced by explainability and visualization methods without sacrificing their quality or usefulness.

## Data Availability

The original contributions presented in the study are included in the article/supplementary material, further inquiries can be directed to the corresponding author.
